# Restoring riparian habitats for benefits to biodiversity and human livelihoods: a systematic map protocol for riparian restoration approaches in the tropics

**DOI:** 10.1186/s13750-025-00355-8

**Published:** 2025-01-30

**Authors:** Sheena Davis, Matthew Grainger, Marion Pfeifer, Zarah Pattison, Philip Stephens, Roy Sanderson

**Affiliations:** 1 Modelling, Evidence and Policy RG, SNES, Newcastle University, Newcastle, NE1 7RU UK; 2 Norwegian Institute for Nature Research, Hogskoleringen 9, NO-7034 Trondheim, Norway; 3 Biological and Environmental Sciences, University of Stirling, Stirling, UK; 4 Department of Biosciences, Durham University, Durham, UK

**Keywords:** Riparian zones, Tropical, Environmental regeneration, Human well-being, Biological diversity, Ecosystem services

## Abstract

**Background:**

Riparian zones are vital transitional habitats that bridge the gap between terrestrial and aquatic ecosystems. They support elevated levels of biodiversity and provide an array of important regulatory and provisioning ecosystem services, of which, many are fundamentally important to human well-being, such as the maintenance of water quality and the mitigation of flood risk along waterways. Increasing anthropogenic pressures resulting from agricultural intensification, industry development and the expansion of infrastructure in tropical regions have led to the widespread degradation of riparian habitats resulting in biodiversity loss and decreased resilience to flooding and erosion. Considering climate change and its associated effects on freshwater systems, the need to build resilience and adaptive capacities is pertinent. This has prompted the need to protect existing riparian habitats and the implementation of solutions to restore these degraded habitats to recover their functional capacity. This systematic map will aim to identify and collate existing literature on approaches for riparian restoration implemented in tropical regions and identify what indicators have been used to measure outcomes for biodiversity and human well-being. The resulting collation of evidence will help to identify current knowledge gaps and inform the direction of future research.

**Methods:**

To address the aims of this systematic map, a search of pre-identified bibliographic databases will be undertaken using a set string of search terms. In addition to this, a grey literature search will be conducted using Google Scholar and by searching for references using specialist websites. All literature that is gathered will be screened by title, abstract and full text using a two-phase screening process which adheres to a pre-determined eligibility criteria. Data will then be coded from the collated group of articles using a pre-designed data coding sheet. Heterogeneity will likely be present in the data; therefore, studies will be grouped appropriately based on the restoration strategy implemented and, on the type of outcome measured. These will be presented as sub-groups. A narrative synthesis of map findings will be produced, this will outline the distribution and frequency of restoration interventions, and outcomes measured, and will highlight evidence gaps to direct future research.

**Supplementary Information:**

The online version contains supplementary material available at 10.1186/s13750-025-00355-8.

## Background

In recent decades land use change in the tropics has accelerated at the fastest rate globally [17] and has spearheaded rates of habitat loss in these regions, whereby tropical forests, for example, have been reduced at a rate of 5.5 M ha/year from years 2010 to 2015 [4]. Largely, agricultural intensification and expansion to meet the increased food demands of a growing human population is one of the main drivers of habitat loss and fragmentation within tropical regions [17, 18]. Additionally, human population growth has led to increases in activities such as wood harvesting for fuel and bush clearing to make way for housing and other infrastructure, further increasing rates of deforestation and the degradation of natural terrestrial and freshwater habitats (Specht et al. 2015), [29]. This has contributed to heightened levels of biodiversity loss due to the reduction in the availability of viable habitats for organisms and consequently, has led to detrimental impacts to continued ecosystem functionality.

Riparian zones, also known as riparian forests or riparian strips, are important transitional habitats between aquatic and terrestrial ecosystems. They provide a wealth of regulating and provisioning ecosystem services and essentially function as a water quality protection buffer by providing important functions such as sediment filtration, pollution retention and erosion control [2, 23]. While these habitats are fundamentally important for maintaining healthy water systems, they also support disproportionally high levels of biodiversity which contributes to a range of biodiversity-regulated ecosystem services. In part, this is due to the role of riparian zones in maintaining connectivity in highly modified landscapes, linking areas of tree cover and thus populations of species, particularly those species that are dependent on tree cover [10]. Furthermore, riparian zones have been shown to play a role in carbon sequestration, as illustrated by Matzek et al. [22], restored riparian sites stored larger quantities of carbon compared to those that were unrestored, indicating their potential for reducing the impacts associated with climate change. In similarity with many natural habitats within the tropics, these transitional zones are subject to increasing degradation due to the pressures placed on these habitats by human encroachment and to a greater extent, agricultural expansion [33].

In the context of agricultural land use, riparian buffer zones play an important role in limiting the runoff of agricultural pollutants such as pesticides, nitrogen, phosphorus, and fine sediments into water sources thus maintaining water quality [5, 30]. Additionally, they help to inhibit the rate of topsoil loss through surface runoff and erosion. Furthermore, the biodiversity supported by these habitats has the potential to offer a range of biodiversity-regulated ecosystem services that would be beneficial to agricultural productivity, such as pest control, pollination, and seed dispersal services [1,9]. Continued degradation of riparian buffer zones could result in loss of ecosystem functionality. Loss of ecosystem function has been shown to have a negative effect on crop yields which has consequently led to higher production costs [27]. In line with Sustainable Development Goal (SDG) 2, promoting high levels of agricultural productivity is considered fundamentally important to reducing poverty levels and ensuring food security, particularly in developing countries [7]. Therefore, finding solutions that allow for continued agricultural productivity while maintaining natural resources and restoring ecosystems from degraded states will be fundamentally important to ensuring positive outcomes for both biodiversity and human well-being.

The protection of existing natural riparian zones and the restoration of degraded riparian zones within agricultural areas have the potential to address concerns around food security and climate change resilience. Studies carried out in tropical agricultural systems have shown that the conservation of natural habitats within the agricultural matrix can have a positive effect on the populations of beneficial species and enhance the potential of the ecosystem services associated with them [9]. As Gonzales-Chaves and Rodolfo [8] illustrated, pollination rates were more frequent in cultivated areas that were in closer proximity to natural habitats, indicating the spillover of beneficial services. Harnessing the biodiversity-regulated ecosystem services provided by riparian habitats within agricultural landscapes will be important for the design of nature-based solutions to allow for more sustainable, cost-effective farming methods. For example, by enhancing natural pest control the need for pesticide application on crops could be reduced [16]. Reducing the need for the application of agricultural inputs will be beneficial to farmers by bringing down their production costs, this is particularly important for small-scale subsistence farming communities that have smaller yield margins in comparison to large-scale industrial producers.

Gaining insights into the application and outcomes of riparian restoration interventions specifically in tropical regions can help to better equip practitioners with a broader knowledge of restoration impacts and how best to prioritise approaches for maximising positive outcomes for both biodiversity and human wellbeing. Largely, research on riparian buffer zones has concentrated on the effectiveness of the services that these habitats can provide [28]. In particular, there has been a focus on their role in providing water filtration, nutrient retention and sedimentation control services for improved water quality [21, 32]. It has been recognised that increased human pressures on these habitats have resulted in degradation and loss of functional capacity, which has prompted an increase in research on restoration approaches for riparian buffer zones over the last 30 years [34], with many interventions focussing on restoring structural aspects of riparian habitats for maintaining ecosystem functionality, such as width of buffer zones [2, 19]. When designing and implementing the restoration of natural habitats, it is also important to consider the potential disservices to human well-being that are associated with improved biodiversity, such as increased pest pressure and incidence of human-wildlife conflicts. Careful trade-off analysis to balance the benefits of restoring natural habitats with the potential disservices and planning for mitigation against these disservices is particularly important in tropical agricultural systems of rural Africa, where crop yields and food and livelihood securities are closely coupled [25]. Therefore, restoration approaches that maximise beneficial services while minimising the effects of disservices should be prioritised.

However, research focused on riparian habitats and their restoration shows a general bias towards temperate regions with noticeably less primary research having been undertaken within tropical regions [6]. As the extent of existing research on restoration activities for riparian habitats in tropical regions is relatively unknown in comparison to temperate regions, a systematic map will provide an opportunity to bring together and present literature on this topic. This systematic map will collate the existing evidence for riparian restoration interventions specifically in the tropics. It will summarise the types of approaches taken, their timescales, the climatic conditions of the study locations, the geographical distribution of research and what indicators have been used to measure outcomes for biodiversity, biodiversity-regulated ecosystem services and subsequently human wellbeing. The collated evidence will be used to produce a knowledge base on restoration activities for riparian habitats within tropical regions, providing restoration practitioners with an overview of interventions and monitoring approaches using indicators of biodiversity and human-wellbeing that could be implemented in the tropics. Furthermore, this systematic map will help to highlight areas where sufficient research has been conducted and would be suitable for a systematic review, as well as key knowledge gaps surrounding riparian restoration in tropical regions where future research can be directed.

### Stakeholder and expert engagement

A brief survey was designed to obtain stakeholder perspectives on riparian restoration interventions and their perceptions of current existing knowledge gaps surrounding the restoration approaches for riparian zones (Supplementary material: Additional file 1). The survey allowed stakeholders to comment on the relevance of the research question following the PICO structure (Table 1), the comprehensiveness of the search strategy and provide feedback for consideration in the protocol, such as alternative search terms and additional databases. This survey was disseminated by email to stakeholders identified through initial literature scoping searches, this included experts from universities, practitioners, and conservation organisations. To date, we have received six responses from stakeholders who have collectively contributed to work on riparian restoration research and interventions in Malaysia, Indonesia, Brazil, and Australia. As part of the search strategy, stakeholders will be contacted to request grey literature, reports and procedural documentation. Aside from their involvement in the searching stage, stakeholders will have no further involvement in the mapping process.

**Table 1:** Key primary question elements following the PICO structure

PICO Element	Description
POPULATION (P)	Riparian habitats in tropical regions globally
INTERVENTION (I)	Restoration approaches (i.e., passive, or active interventions /engineered interventions or nature-based interventions)
COMPARATOR (C)	Before and after intervention at the study site or restored sites versus unrestored sites
OUTCOME (O)	Measurable indicator of biodiversity such as species richness, species abundance, a population change of a selected indicator species
	Measurable indicator of human well-being in the context of material wealth, health, and security, such as water quality, pollinator quantity or diversity, crop quality and quantity, soil quality

Following the circulation of the survey, stakeholders highlighted the need to develop suitable indicators to determine the effectiveness of riparian restoration interventions which can be used for long-term monitoring post-restoration. The aim of this systematic map to identify indicators for outcomes for biodiversity and human well-being following riparian restoration will therefore be an important first step in outlining what indicators have already been used and future research can be aimed at determining how appropriate these indicators are for long-term monitoring purposes. In the interim, the indicators identified through this mapping exercise can provide stakeholders with an overview of potential options that could be considered for future post-restoration monitoring procedures.

## Objectives of the review

This systematic map aims to collate evidence from existing literature to provide an overview of restoration approaches implemented in the tropics to aid the protection and recovery of riparian habitats specifically for outcomes for biodiversity and human-wellbeing. This systematic map aims to describe evidence on all restoration approaches that have been implemented, i.e., passive or active, nature based or hard engineering solutions. It will also look to identify measurable indicators for biodiversity and human well-being outcomes which could be used for monitoring purposes. For biodiversity outcomes, this encompasses indicators such as species richness and abundance. Human well-being outcomes encompass indicators related to ecosystem services associated with three of the five human well-being domains specified by Loveridge et al. [20]. The chosen domains include material well-being, health, and security as they can be linked directly to ecosystem services provided by riparian buffers. For example, ecosystem services such as sediment filtration can be directly associated to human health. The evidence that will be collated through the production of this systematic map will provide an overview of the current understanding of the implementation and monitoring of riparian restoration interventions in tropical regions. It will identify areas where more research needs to be directed. Finally, the collated evidence will be used to guide the research design of a prospective project looking to establish a procedure for decision-making for riparian restoration and on-going monitoring in rural tropical landscapes.

### Primary question

What evidence exists on riparian zone restoration interventions in tropical regions specifically in terms of outcomes for biodiversity and human well-being?

### Secondary questions

The primary question that this systematic map aims to address is broad and aims to encompass all interventions that have been implemented in tropical regions. Therefore, to fully address the primary research question the following secondary questions will be answered.

1. What are the characteristics of restoration interventions that have been used in tropical regions—intervention type, geographical scale, timescale, and climate?
2. What elements of the riparian zone have been the focus of the restoration intervention (i.e., vegetative elements such as trees and reed banks, the extent of buffer width or structural elements such as riverbanks)?
3. What biodiversity indicators have been used to assess or monitor restoration interventions?
4. What human well-being indicators have been used to assess or monitor restoration interventions?

The primary question was broken down into four main components following the PICO structure (Population, Intervention, Comparator and Outcome) outlined in Tables 1 and 2.

**Table 2:** Search terms for each PICO element and the resultant final search string

PICO Element	Search terms
POPULATION (P)	riparia*^a^ OR floodplain$ OR creek$ OR stream$ OR river* OR wetland$ OR lake$
INTERVENTION (I)	restor* OR reclaim* OR regen* OR “re-establish*”, OR renew* OR reveg* OR rehabilitation OR reconstruct* OR replant* OR engineer* OR protect* OR “set-aside” OR remediat* OR revitaliz* OR “de-canalisation”, OR “re-meandering”, OR “re-wetting”, OR reforest*
OUTCOME (O)	biodivers* OR divers* OR "ecosystem service$", OR "ecological service$", OR “ecological function$", OR “ecosystem function$”,OR "species richness", OR “species abundance", OR pollination OR “pest control”, OR “ seed dispersal”, OR “carbon sequestration”, OR “carbon storage”, OR “natural capital", OR "water quality", OR "crop yield$", OR "crop production", OR connectivity OR resilien* OR flood$ OR erosion OR "bank stability", OR "flood protection", OR "flood mitigation", OR "human health", OR "food security", OR nutrition OR safety
LOCATOR	tropic* OR “global south", OR "southern hemisphere",
SEARCH STRING	riparia* OR floodplain$ OR creek$ OR stream$ OR river* OR wetland$ OR lake$ AND restor* OR reclaim* OR regen* OR “re-establish*”, OR renew* OR reveg* OR rehabilitation OR reconstruct* OR replant* OR engineer* OR protect* OR “set-aside”, OR remidiat* OR revitaliz* OR “de-canalisation”, OR “re-meandering”, OR “re-wetting”, AND biodivers* OR divers* OR "ecosystem service$", OR "ecological service$", OR “ecological function$", OR “ecosystem function$”, OR "species richness", OR “species abundance", OR pollination OR “pest control”, OR “seed dispersal”, OR “carbon sequestration”, OR “carbon storage”, OR “natural capital", OR "water quality", OR "crop yield$", OR "crop production", OR resilien* OR flood$ OR erosion OR "flood protection", OR "flood mitigation", OR "human health", OR "food security", OR nutrition OR safety AND tropic* OR "global south", OR "southern hemisphere",

^a ^ The symbol [*] is used here to broaden the search allowing for the return of variations of that term

## Methods

The protocol for this systematic map follows the guidelines set out by the Collaboration for Environmental Evidence (CEE) Guidelines and Standards for Evidence Synthesis in Environmental Management [3] and adheres to the ROSES reporting standards (see [13], Supplementary material, Additional file 2). The development of the search strategy and search strings for this review was undertaken in collaboration with an information specialist (library liaison at Newcastle University—Julia Robinson).

### Search strategy

To identify relevant literature a comprehensive search which will be inclusive of articles, theses, books and grey literature will be conducted. This will be undertaken using pre-identified databases and where necessary the full texts of relevant papers will be requested directly from the authors. Relevant review articles will also be searched to obtain references for articles that could be applicable. The following publication databases will be searched systematically: Web of Science Core Collection, Scopus, CAB Abstracts, and ProQuest Natural Science Collection. Of these identified databases we expect that the majority of relevant literature will be sourced from Web of Science Core Collection and Scopus. 12 benchmark articles were chosen through preliminary reading and initial scoping searches that provide examples of riparian restoration interventions in tropical landscapes. These benchmark articles were used to test the comprehensiveness of the search string (Supplementary material: Additional file 3).

To further enhance the comprehensiveness of this systematic map, supplemental searches using the R-based tool; *citationchaser* [14], which allows for forward and backward citation chasing, will be used to identify relevant literature that the initial database searches may have missed. To search for supplementary grey literature, a web-based search using the search engine Google Scholar will be carried out, additionally a specialist website namely, ‘The Applied Ecology Resources’ will be searched to obtain any references to relevant documents that may have been missed through database searches alone. Stakeholders and any expert groups identified through this review will be contacted to request relevant articles, reports, and any procedural documents on riparian restoration. The International Union for the Conservation of Nature and the organisation World Agroforestry (ICRAF) have already been identified and will be contacted to request any relevant literature.

### Search terms

The search question was broken down into three main elements: population, intervention, and outcome. Key terms and their synonyms as well as common phrases associated with these elements were identified. These were then used to build a search string, using ‘OR’ between each key term and ‘AND’ to combine these three main elements. This allowed for articles to be identified which referred to the restoration of riparian habitats and were inclusive of a measure of a relevant biodiversity metric and/or a measure of human well-being. The locator terms ensured that only studies in tropical regions would be returned. Following consultation with experts and stakeholders several additional search terms were suggested, of these, the following have been included in the final search string, ‘de-canalisation,’ ‘re-meandering’ and ‘re-wetting’ (intervention) and ‘connectivity,’ ‘water yield,’ and ‘bank stabili*,’ (outcome).

The final search string was adjusted to fit the syntax of each database. The testing of the search string for optimisation was carried out in Web of Science against their Core Collection, using the 12 benchmark articles to gauge the comprehensiveness of the search string. A final search using the final search string was carried out in Web of Science against their CORE collection and in Scopus. The search string generated 2976 returns in Web of Science, this included 11 of the 12 benchmark articles. The final search carried out in Scopus generated 3357 returns which included 9 of the 12 benchmark articles. While the number of returns generated is relatively low, confirming the expected bias of research directed towards temperate regions, the implementation of a systematic map is relevant to ascertain the current literature base that exists for tropical regions. The final search string will be used to search the pre-defined publication databases, specialist websites and web search engines for all available literature. No timeframe constraint or language constraint will be imposed on the searches. This is to ensure that all available literature is obtained and is inclusive of studies from non-English speaking regions which would otherwise be overlooked. Where studies are returned in another language a translator who will be able to translate the article to English will be sought out. It is acknowledged that the decision to conduct the search in English may omit relevant articles that are in another language however multiple language searches are not possible due to resource constraints.

### Article screening and study eligibility criteria

#### Screening process

A screening team, consisting of the primary reviewer and two or three secondary reviewers (depending on the quantity of literature returned) will evaluate the returned eligible literature by using an inclusion/ exclusion criterion (see Eligibility Criteria below). All studies that enter the filtering process will undergo a double screening at each filtering stage, to adhere to best practice guidelines. The primary reviewer will lead the screening process and undertake the entirety of the process while secondary reviewers will be assigned subsets of the returned literature and will conduct independent screenings of these articles in parallel with the primary reviewer. This will ensure that all articles are independently screened by the primary reviewer and one other reviewer.

A ROSES flow diagram will be created using the ROSES flowchart R package, this diagram conforms to the ROSES reporting standards for systematic maps and reviews and will be used to illustrate the quantity of papers that will be excluded at each stage of the screening process [11]. To begin the screening process the collated library of articles will be checked for duplicates using the ‘ASyD’ R package [15], which will clean the library of duplicates by identifying title and DOI matches. Once the duplicates have been removed from the library it will then undergo the filtering process. The two-stage filtering process will be carried out using Rayyan [26], as this will allow articles to be screened simultaneously by members of the review team. The first stage of the filtering process will filter articles based on title and abstract using the pre-defined eligibility criteria shown below to guide decision-making on whether to include an article. Where there is uncertainty regarding an article’s inclusion based on the title and abstract, i.e., there is insufficient information detailed within the title and abstract to make a conclusive decision, then it will be carried forward to the next stage of the filtering process. While the full-text filtering process will be carried out in Rayyan, all full-text articles will be stored separately in a reference management tool called Zotero. During the filtering process reviewers will tag articles with labels showing the decision, these are: ‘Include’, ‘exclude’, and ‘maybe’. Meetings between the primary reviewer and secondary reviewer will be held at 30%, 60%, and 100% completion of the 1st stage of the filtering process (abstract and title screening), and again during the 2nd stage of the screening process (full-text screening). To ensure that the eligibility criteria is robust and replicable the consistency of decision making during both filtering stages will be assessed using Cohen’s Kappa test which is used to calculate the proportional agreement between two reviewers. A resulting kappa value greater than 0.6 indicates consistency and accuracy between reviewers and gives an indication of the overall replicability of the eligibility criteria. During the reviewer meetings, articles with a decision conflict will be discussed to reach a final judgement on the inclusion or exclusion of the article. Where titles have been tagged as ‘maybe’ in the first filtering stage, these will be carried forward to the next filtering stage. Any articles that are still tagged as maybe by the end of the final filtering stage will be reviewed by all members of the team to reach a final consensus. It is anticipated that none of the reviewers conducting the filtering process for this systematic map will have authored any articles that may be returned. However, in the unlikely event that this is found to be the case this will be made known and all decisions regarding the inclusion or exclusion of the article will be explicitly reported.

#### Eligibility criteria

The literature returned from the searches will be collated. They will then undergo two stages of filtering. Stage 1; Title and Abstract filtering and Stage 2; Full text filtering. To filter the literature a set eligibility criterion, detailed below, will be used to determine whether the study is relevant and should be included within the systematic map. A list of all articles excluded during the second stage of filtering along with reasons for their exclusion in line with the eligibility criteria will be provided.

1. Population: This study will look at all riparian habitats in tropical regions globally. Tropical regions in this study refer to all geographical areas located within the tropics. Riparian habitat in the context of this study is defined as a transitional zone between the aquatic and terrestrial environment, these are also referred to as riparian buffers, riparian strips, or vegetative strips. For the purposes of this study a riparian zone must be located adjacent to any freshwater body, such as rivers, streams, creeks, lakes and wetlands.

- Exclude if the study is not explicitly located within the tropics, here defined any location between the latitudes of 23.5° north and 23.5° south.
- Exclude if the study does not refer to a body of freshwater to which the riparian zone is in proximity.
- Exclude if the study refers to mangroves as these are not included within this systematic mapping exercise.

2) Intervention: This study aims to collate evidence on approaches implemented to restore degraded riparian habitats. Restoration approaches include any intervention aimed at restoring, improving or protecting any aspect of a riparian habitat. This may include hard-engineering solutions aimed at improving bank stability, nature-based approaches, restoring elements of a riparian habitat such as planting trees or reed banks and through approaches aimed at setting aside protected riparian areas.

- Exclude if no explicit mention of any restoration activity.

3) Outcome: This systematic map specifically looks to collate evidence on riparian restoration specifically for outcomes related to biodiversity and human-wellbeing. This will encompass any indicator used to measure biodiversity or an element of human well-being. For biodiversity, indicators include but are not limited to species richness, species abundance, species specific population dynamics, occurrence and establishment of invasive species and genetic variation. Human well-being outcomes will include indicators related to ecosystem services within three of the five domains of human well-being identified by Loveridge et al. [20]: material well-being, health, and security. For example, ecosystem services provided by riparian vegetative buffers such as sediment filtration and nutrient retention can positively impact human health by improving water quality [24], therefore indicators relating to water quality will be relevant for inclusion. Similarly, pollination services can contribute to higher crop yields thus improving food security [31].

- Exclude if the study does not explicitly mention any indicator related to biodiversity or human wellbeing.
- Exclude if the indicator for human wellbeing does not directly relate to any of the three human wellbeing domains: material well-being, health or security.

4) Comparator: This systematic map will collate studies where a comparison has been made. This includes a comparison of before and after a restoration intervention, a comparison of unrestored versus restored sites or an experimental protocol that includes a comparison of intervention versus control.

- Exclude if no comparison between pre and post restoration is made
- Exclude if experimental design does not include a control.
- Exclude if no comparison between a restored and unrestored site.
- Study Type: This systematic map will focus on primary research conducted on riparian restoration interventions. Reviews of restoration interventions will be considered as secondary research and will not be eligible for inclusion in this systematic map.
- Exclude if the study is a review of an intervention and not primary research.

### Study validity assessment

The objective of this systematic map is to provide a broad and detailed overview of the existing evidence base for riparian restoration interventions in the tropics, giving insight into the geographic distribution and topical extent of the evidence base. As such, a formal assessment of study validity will not be conducted beyond the eligibility criteria described above.

### Data coding strategy

For each study that has met the eligibility criteria and is accepted after the full-text screening, a reviewer will code data regarding the study design, location and climate characteristics, timescale, type of restoration intervention and the outcomes measured. Outcome data will include type of outcome measured, i.e., relating to biodiversity or human-wellbeing and type of indicator used, e.g., species richness for biodiversity or water quality measures for human-wellbeing. Details of each study will be extracted and compiled to a pre-designed data coding form (Supplementary Material: Additional file 4). Where there is multiple separate restoration interventions implemented in the same article, for example, if the article looked to compare restoration approaches, then these will be treated as individual studies and listed as individual records in the data coding form.

Prior to the data coding of the final articles being carried out, the data coding form will be piloted independently by two members of the review team using the 12 benchmark articles identified during preliminary reading and scoping searches. This will ensure that the components of the data coding table fully encapsulate all relevant information from the articles. Where there are discrepancies then the data coding form will be adjusted to accommodate any additional information that is identified during the pilot coding. Each member of the review team will receive a subset of the final articles from which they will code the required information. Records will then be cross-checked independently by another review team member to ensure that the data coding strategy is replicable and that final records are accurate ensuring all key information from each article has been coded. If data is unavailable or incomplete, then an attempt will be made to contact the authors of the article to obtain the missing information. Where there is uncertainty in data extraction, this will be discussed with the second reviewer. Where uncertainty persists then the entire review team will consult, and a consensus will be reached. Any modifications to the draft version of the data coding form and reasons for changes will be reported, furthermore, all data that is coded from the articles will be provided as an appendix alongside the final publication.

### Study mapping and presentation

A narrative synthesis of all the studies included after the two-phase screening process will be produced. This will include tables, figures, and descriptive statistics that support the interpretation of results. It will include a summary of each study's intervention, context, study design, and reported indicators. It is anticipated that the data coded from the final group of included studies is likely to have high levels of heterogeneity in terms of different restoration interventions used, the type of indicators used, and the type of study design. The coded data from articles will therefore be grouped appropriately, following discussions with the review team. It is anticipated that they will be grouped by the type of restoration intervention that is implemented and whether the indicator used relates to biodiversity outcomes which, for example, may include species richness or species abundance or human well-being indicators which, for example, may include water quality or crop yields. A table and/or diagram will be used to present the indicators related to each study and the intervention type. A map of the final evidence base collated through the review will be compiled using the existing tool; EviAtlas [12]. This tool will be used to present the geographic distribution and number of studies undertaken in tropical regions. This will include the climatic characteristics of each location as stated in the study (e.g., average rainfall per annum, the duration of the ‘wet’ season and average annual temperature range), the type of intervention that has been carried out for each study, the outcomes that have been reported and the timescales associated with the intervention. This geographic map will illustrate the tropical areas where the most research on restoration has been conducted and will highlight regions where little to no research has been conducted, thus, identifying areas where more research should be directed. This systematic mapping exercises may also reveal areas where there is sufficient existing evidence where a future systematic review may be applicable. Upon publication of this systematic map, a data file of all literature screened will be made available with a permanent Digital Object Identifier (DOI) to ensure preservation and access. This will include the reasons for exclusion at the full-text filtering stage and the meta-data associated with each article included following the two-stage filtering process.

## Supplementary Information

Additional file 1: A copy of the brief survey that was distributed to stakeholders

Additional file 2: ROSES Systematic map protocol form

Additional file 3: List of benchmark articles used to test the comprehensiveness of the search string

Additional file 4: Pre-designed data coding form

## Data Availability

Data sharing is not applicable as no datasets were generated or analysed to produce this protocol.
