# Supplementary material for: Restoring riparian habitats for benefits to biodiversity and human livelihoods: a systematic map protocol for riparian restoration approaches in the tropics
Source: Environ Evid. 2025 Jan 30;14:2. doi: 10.1186/s13750-025-00355-8 (PMC11780855; doi:10.1186/s13750-025-00355-8)
Supplement: Supplementary file 1 — Additional file 1: A copy of the brief survey that was distributed to stakeholders [file 13750_2025_355_MOESM1_ESM.docx]

**Supplementary Material: Additional File 1**

Search strategy survey disseminated to stakeholders in the field of riparian restoration.

**Riparian Zone Restoration Approaches in Tropical Regions**

**Stakeholder Survey**

**INTRODUCTION:**

The team plans to produce a systematic map around the research question “What evidence exists on riparian zone restoration interventions in tropical regions specifically in terms of outcomes for biodiversity and human well-being? ” We aim to collate evidence to provide an overview of restoration approaches implemented in the tropics to aid the protection and recovery of riparian habitats specifically for outcomes for biodiversity and human well-being. This systematic map aims to collate evidence on all restoration approaches that have been implemented, i.e., passive or active, nature-based or hard engineering solutions.  It will also look to identify measurable indicators for biodiversity and human well-being outcomes which could potentially be used for monitoring purposes. Human well-being outcomes encompass indicators related to improved ecosystem services of three of the five human-wellbeing domains specified by Loveridge *et al.,* (2020); these are material well-being, health, and security. From the results, we aim to inform the design of a cost-effective restoration process that focuses on riparian habitats in rural tropical landscapes and accounts for equity, social and biodiversity outcomes.

We plan to search all relevant literature using a systematic protocol. As stakeholders, we kindly ask for your feedback on and insights into the topic and the search strategies. This will help to minimize bias and increase the transparency of this process.

Please note that all personal data gathered through this survey will be stored securely and will remain confidential. The responses gathered through this survey will be used for research purposes only and will not be published without prior consent (see section IV).

Should you require any further information regarding data privacy, or any other aspect of this survey please contact; Sheena Davis ([S.M.Davis2@newcastle.ac.uk](mailto:S.M.Davis2@newcastle.ac.uk)).

Thank you for your participation in this survey.

1. **Respondent details**

Name:

Email:

Affiliation:

Position/Occupation:

Do you consider yourself an expert and/or stakeholder in the context of riparian zone restoration?

Could you briefly explain why?

Are there specific regions where you have researched riparian habitats or contributed to riparian restoration interventions?

1. **Topic Exploration**
2. Could you briefly outline 1-4 of the most pressing research questions or subtopics regarding riparian zone restoration in tropical regions that you believe require more research?
3. Based on your experience and knowledge of riparian zones and their importance for maintaining biodiversity and ecosystem services, what indicators of increased biodiversity and improved human well-being would you consider to be most relevant to riparian restoration in tropical regions?
4. Based on your knowledge, what interventions, whether hard engineering solutions or soft nature-based solutions, for riparian restoration, have been implemented in tropical regions or elsewhere?
5. **Search strategy**
6. Are there other sources of information (databases, organizational websites) you suggest we include in our search beyond those listed below?

ProQuest: Natural Sciences Collection

Web of Science

Scopus

CAB Abstracts

1. Listed below are the search terms we plan to use to find relevant literature. Are there any additional search terms or modifications you would use if you were conducting the literature search?

| **Elements** | **Search terms** |
| --- | --- |
| Population | Riparian river*  Riparian buffer* wetland*  Riparian zone* stream*  Riparian habitat* creek*  Riparian woodland*  Riparian forest*  Riparian strip*  Riparian corridor*  Riparian ecosystem*  Riparian rangeland* |
| Intervention | restor* protect*  regen* set-aside*  reveg* engineer*  replant*  remed*  reclaim*  renew*  recontruct*  re-establish* |
| Biodiversity Outcomes | biodivers* ecological service*  divers*  species richness  species abundance  ecosystem service*  ecological function* |
| Human-wellbeing Outcomes | resource access livestock health  economic gain erosion control  economic growth  natural capital  water quality  water health  crop yield*  crop production  security  resilien*  flood protection  human health |

* Indicates searching for word variations (e.g., river* can be rivers or riverine)

1. **Data privacy**

Do you allow us to identify you as a stakeholder in the systematic map publication?

___ Yes ___ No

May we share your responses in an aggregate summary in the systematic map publication?

___ Yes ___ No ___ Please contact me first
