# Supplementary material for: Restoring riparian habitats for benefits to biodiversity and human livelihoods: a systematic map protocol for riparian restoration approaches in the tropics
Source: Environ Evid. 2025 Jan 30;14:2. doi: 10.1186/s13750-025-00355-8 (PMC11780855; doi:10.1186/s13750-025-00355-8)
Supplement: Supplementary file 3 — Additional file 3: List of benchmark articles used to test the comprehensiveness of the search string [file 13750_2025_355_MOESM3_ESM.docx]

**Supplementary Material: Additional File 3**

List of 12 Benchmark articles

1. Suganuma MS, Torezan JMD, Durigan G. Environment and landscape rather than planting design are the drivers of success in long-term restoration of riparian Atlantic forest. *Appl Veg Sci*. 2018;21(1):76-84.
2. Manrique-Hernández H, Heartsill-Scalley T, Barreto-Orta M, Betancourt-Román CM, Ortiz-Zayas JR. Assessing restoration outcomes in light of succession: management implications for tropical riparian forest restoration. *Ecol Restor*. 2016;34(2):147-158.
3. Calle A, Holl KD. Riparian forest recovery following a decade of cattle exclusion in the Colombian Andes. *For Ecol Manag*. 2019;452:117563. doi: 10.1016/j.foreco.2019.117563.
4. De Paula FR, Ruschel AR, Felizzola JF, Frauendorf TC, de Barros Ferraz SF, Richardson JS. Seizing resilience windows to foster passive recovery in the forest-water interface in Amazonian lands. *Sci Total Environ*. 2022;828:154425.
5. Mello K de, Randhir TO, Valente RA, Vettorazzi CA. Riparian restoration for protecting water quality in tropical agricultural watersheds. *Ecol Eng*. 2017;108:514-524.
6. Novais JMP, Ramos FT, Dores EFG de Carvalho, Maia JC de S. Impact of different revegetation techniques on soil and plant attributes in a riparian zone. *Caatinga*. 2020;33(1):151.
7. Lisboa T de FB, Cielo-Filho R, Câmara CD. Applicability of monitoring protocols developed for active restoration projects in the evaluation of passive restoration of a subtropical riparian forest in Brazil. *Trop Ecol*. 2021;62(1):17-26.
8. Londe V, De Sousa HC, Kozovits AR. Litterfall as an indicator of productivity and recovery of ecological functions in a rehabilitated riparian forest at Das Velhas River, southeast Brazil. *Trop Ecol*. 2016;57(2):355-360.
9. Cortez-Silva EE, Santos G de S, Leite MG de P, Eskinazi-Sant’Anna EM. Response of cladoceran assemblages to restoration of riparian vegetation: a case study in a tropical reservoir of Brazil. *Limnologica*. 2020;85:125822.
10. Suganuma MS, Durigan G. Indicators of restoration success in riparian tropical forests using multiple reference ecosystems. *Restor Ecol*. 2015;23(3):238-251.
11. Espinoza-Toledo A, Mendoza-Carranza M, Castillo MM, Barba-Macías E, Capps KA. Taxonomic and functional responses of macroinvertebrates to riparian forest conversion in tropical streams. *Sci Total Environ*. 2021;757:143972.
12. van Meerveld I, Jones JPG, Ghimire CP, Zwartendijk BW, Lahitiana J, Ravelona M, et al. Forest regeneration can positively contribute to local hydrological ecosystem services: implications for forest landscape restoration. *J Appl Ecol*. 2021;58(4):755-765. Epub 2021 Feb 22.
